# Clinicopathological Features of Thyroid-Like Low-Grade Nasopharyngeal Papillary Adenocarcinoma: A Case Report and Review of the Literature

**DOI:** 10.3389/fsurg.2020.596796

**Published:** 2020-11-19

**Authors:** Hiromasa Takakura, Takeru Hamashima, Hirohiko Tachino, Akira Nakazato, Hiroshi Minato, Masakiyo Sasahara, Hideo Shojaku

**Affiliations:** ^1^Department of Otorhinolaryngology, Head and Neck Surgery, Faculty of Medicine, Academic Assembly, University of Toyama, Toyama, Japan; ^2^Department of Pathology, Faculty of Medicine, Academic Assembly, University of Toyama, Toyama, Japan; ^3^Department of Diagnostic Pathology, Ishikawa Prefectural Central Hospital, Kanazawa, Japan

**Keywords:** thyroid transcription factor-1, thyroglobulin, endoscopic resection, clinicopathological features, thyroid-like low-grade nasopharyngeal papillary adenocarcinoma

## Abstract

Thyroid-like low-grade nasopharyngeal papillary adenocarcinoma (TL-LGNPPA) is an extremely rare neoplasm of the nasopharynx. Accordingly, its clinical and pathological characteristics are not well-known. We report a case of TL-LGNPPA and review the relevant literature on TL-LGNPPA. A 38-year-old Japanese woman presented with a history of nasal obstruction that had persisted for 1 month after symptoms of a common cold (e.g., low-grade fever, sore throat, and fatigue). A pedunculated tumor of ~20 mm in diameter was found on the posterior edge of the nasal septum. The tumor was endoscopically resected. Based on careful histopathological and immunohistochemical examinations, it was diagnosed as TL-LGNPPA. At 5 years after surgery, the patient remained disease-free. TL-LGNPPA has a very good prognosis, and complete resection with a sufficient safety margin is recommended as the first-line treatment. The morphological characteristics and immunohistochemical findings, especially TTF-1 positivity and thyroglobulin negativity, are important for the diagnosis.

## Introduction

Primary nasopharyngeal adenocarcinomas (NPACs) are rare neoplasms, accounting for only 0.38–0.48% of all malignant nasopharyngeal neoplasms (1, 2). NPACs are classified into 2 main groups based on marked differences in their clinical manifestations, histomorphological features, and behavior: the surface origin type, which is usually papillary in configuration and of low-grade malignancy, and the salivary gland type (3). In 1988, Wenig et al. first described low-grade nasopharyngeal papillary adenocarcinomas (LGNPPAs) that had an indolent clinical behavior and low-grade histological features as a distinct entity (4). LGNPPA is histologically characterized by papillary fronds and crowded glandular structures lined by a bland cuboidal to columnar epithelium resembling papillary thyroid carcinoma. In 2005, Carrizo and Luna first reported that two pediatric patients with LGNPPA showed the expression of thyroid transcription factor-1 (TTF-1) in their tumor cell nuclei and named LGNPPA with the expression of TTF-1 “thyroid-like low-grade nasopharyngeal papillary adenocarcinoma” (TL-LGNPPA) (5). TL-LGNPPA is an extremely rare tumor; to date, only 27 cases have been reported (6).

We herein report the case of a 38-year-old Japanese woman with primary TL-LGNPPA and review the relevant literature on TL-LGNPPA.

## Case Report

**Figure 1:**
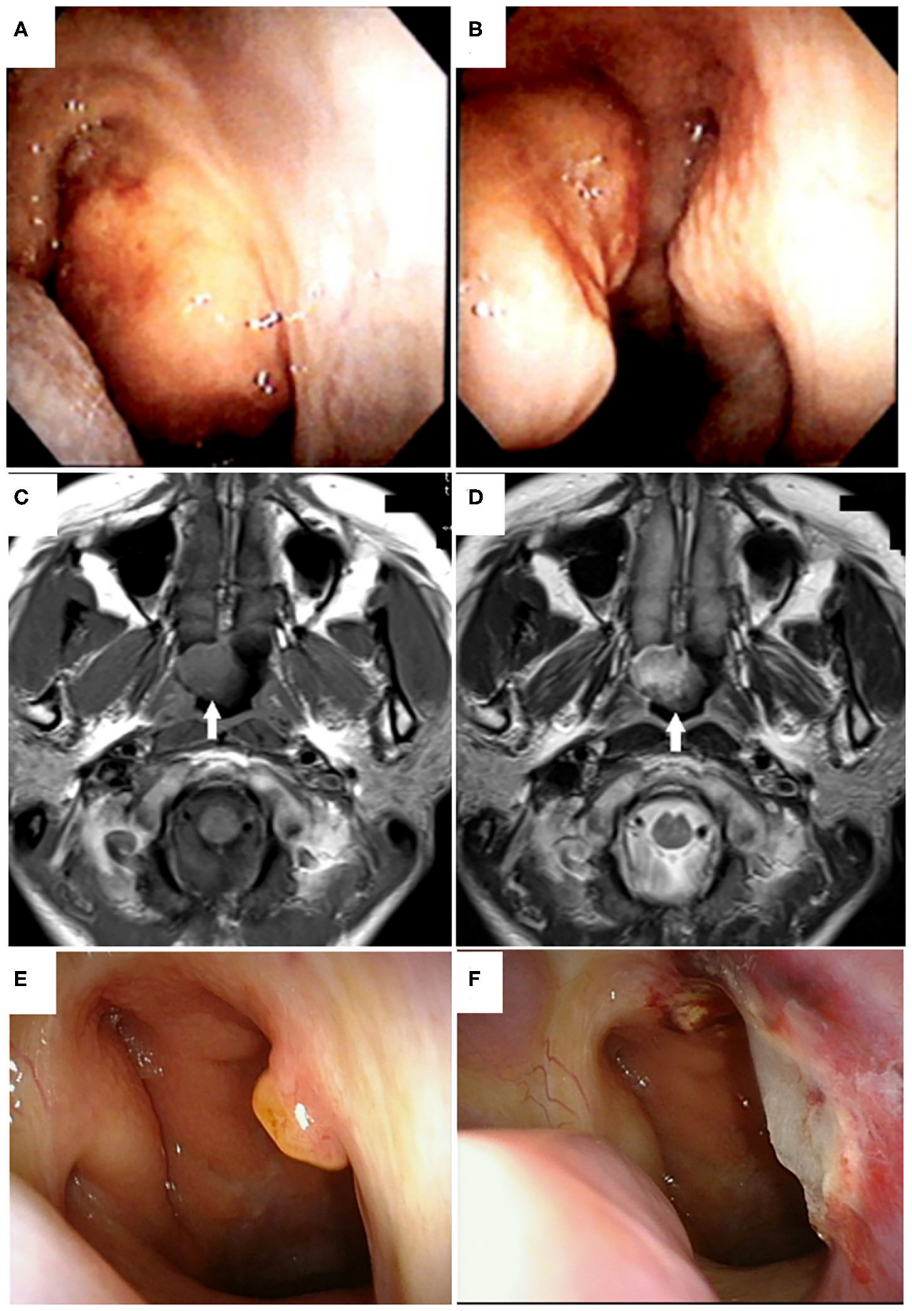
Tumor appearance on nasopharyngeal endoscopy and magnetic resonance imaging (MRI). Endoscopic findings of the nasopharyngeal tumor viewed from the right **(A)** and left **(B)** nasal cavities at the first visit to the previous hospital are shown. A pedunculated polypoid tumor originating from the posterior edge of the nasal septum was found in the epipharynx. Horizontal views of plain T1-weighted **(C)** and T2-weighted magnetic resonance imaging **(D)** of the head showed a tumor of ~20 mm in diameter located in the epipharynx originating from the posterior edge of the nasal septum without invasive or destructive findings (white arrows). T1- and T2-weighted images showed the same or slightly higher intensities compared to that of the nasal concha. Preoperative **(E)** and postoperative **(F)** endoscopic findings of the nasopharyngeal tumor viewed from the right nasal cavity in our hospital. In the preoperative view, the main portion of tumor had disappeared and only the pedunculated portion remained **(E)**. The tumor was endoscopically resected with a 5-mm safety margin **(F)**.

A 38-year-old Japanese woman with a previous history of urticaria caused by an unknown allergen presented to a hospital in 2015 with nasal obstruction that had persisted for 1 month after symptoms of the common cold (e.g., low-grade fever, sore throat, and fatigue). An inspection of the nasal cavity with a soft fiberscope revealed a pedunculated polypoid tumor of ~20 mm in diameter on the posterior edge of nasal septum (Figures 1A,B). Magnetic resonance imaging (MRI) revealed a 20-mm tumor located in the epipharynx that originated from the posterior edge of the nasal septum, and T1- and T2-weighted images showed the same or slightly higher intensities compared to that of the nasal concha (Figures 1C,D). A chest X-ray examination showed no signs of a lung lesion. Enhanced computed tomography (CT) or MRI was not performed because of the patient's history of allergy due to an unknown allergen. A clinical examination revealed no signs of thyroid tumor, cervical lymphadenopathy, or other physical abnormalities. A biopsy of the pedunculated portion of the mass was performed, and it was diagnosed as a benign salivary gland-type tumor. The patient was referred to our hospital and presented for surgical treatment 2 months after first visiting the previous hospital in 2015. On the first inspection of the nasopharynx in our hospital, the main part of the tumor had disappeared and only the pedunculated portion of the tumor remained (Figure 1E). Plain CT revealed no invasive findings or metastatic lesions. The tumor was endoscopically resected 3 weeks after the patient's first visit to our department. In this operation, the tumor was completely excised with a surgical margin of ~5 mm using a needle electrode knife and was removed together with the periosteum from the vomer (Figure 1F).

**Figure 2:**
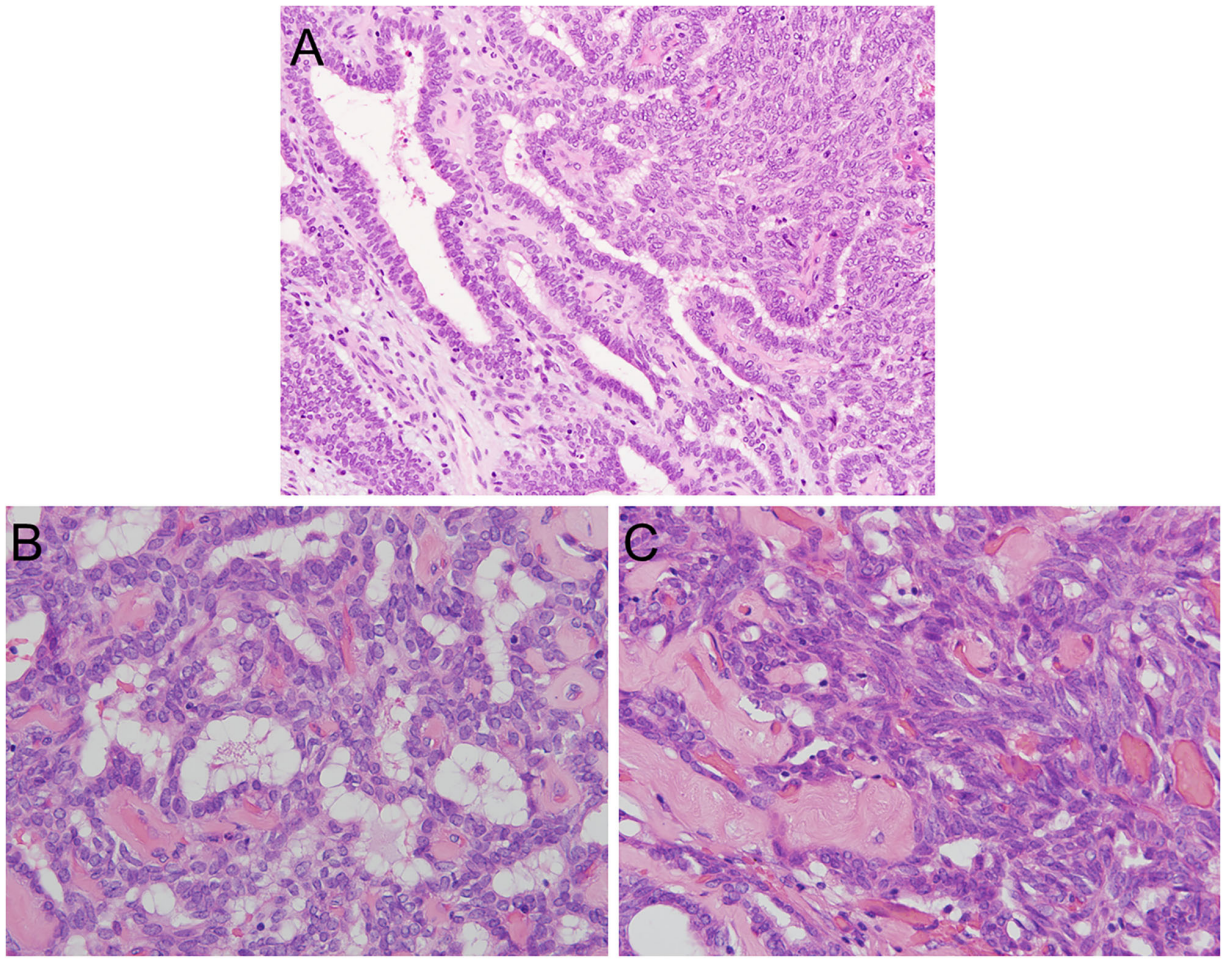
Histopathological features of TL-LGNPPA. **(A)** Histological examination revealed a papillary structure with fibrovascular cores lined by cuboidal to columnar stratified cells with round to oval vesicular nuclei and eosinophilic cytoplasm. Psammoma bodies were not seen. The tumor showed invasive growth into the underlying fibrous connective tissue. (H&E staining, × 20). **(B)** An increase in nuclear chromatin and mild nuclear atypia were found, but no nuclear polymorphism was detected. Some cells had clear chromatin; however, the nuclear groove and nuclear pseudoinclusion were absent. No mitotic figures were found, and necrosis was not identified (H&E staining, × 40). **(C)** A streaming pattern lining of the tumor cells was also found in some areas (H&E staining, × 40).

A histologic examination revealed a papillary structure with hyalinized fibrovascular cores lined by cuboidal to columnar stratified cells with round to oval vesicular nuclei and eosinophilic cytoplasm (Figure 2A). An increase in nuclear chromatin and mild nuclear atypia were found, but no nuclear polymorphism was detected. Some cells had clear chromatin; however, a nuclear groove and nuclear pseudoinclusion were absent. No mitotic figures were found and there was no necrosis (Figure 2B). A streaming pattern lining of the tumor cells with small round to oval nuclei (i.e., spindle cell component) was also found in some areas (Figure 2C). Psammoma bodies were not seen. The tumor showed invasive growth into the underlying fibrous connective tissue (Figure 2A). These morphological findings suggested polymorphous low-grade adenocarcinoma (PLGA) and low-grade nasopharyngeal papillary adenocarcinoma (LGNPPA) as differential diagnoses. Additional immunohistochemical examinations were needed to make a definitive diagnosis.

**Figure 3:**
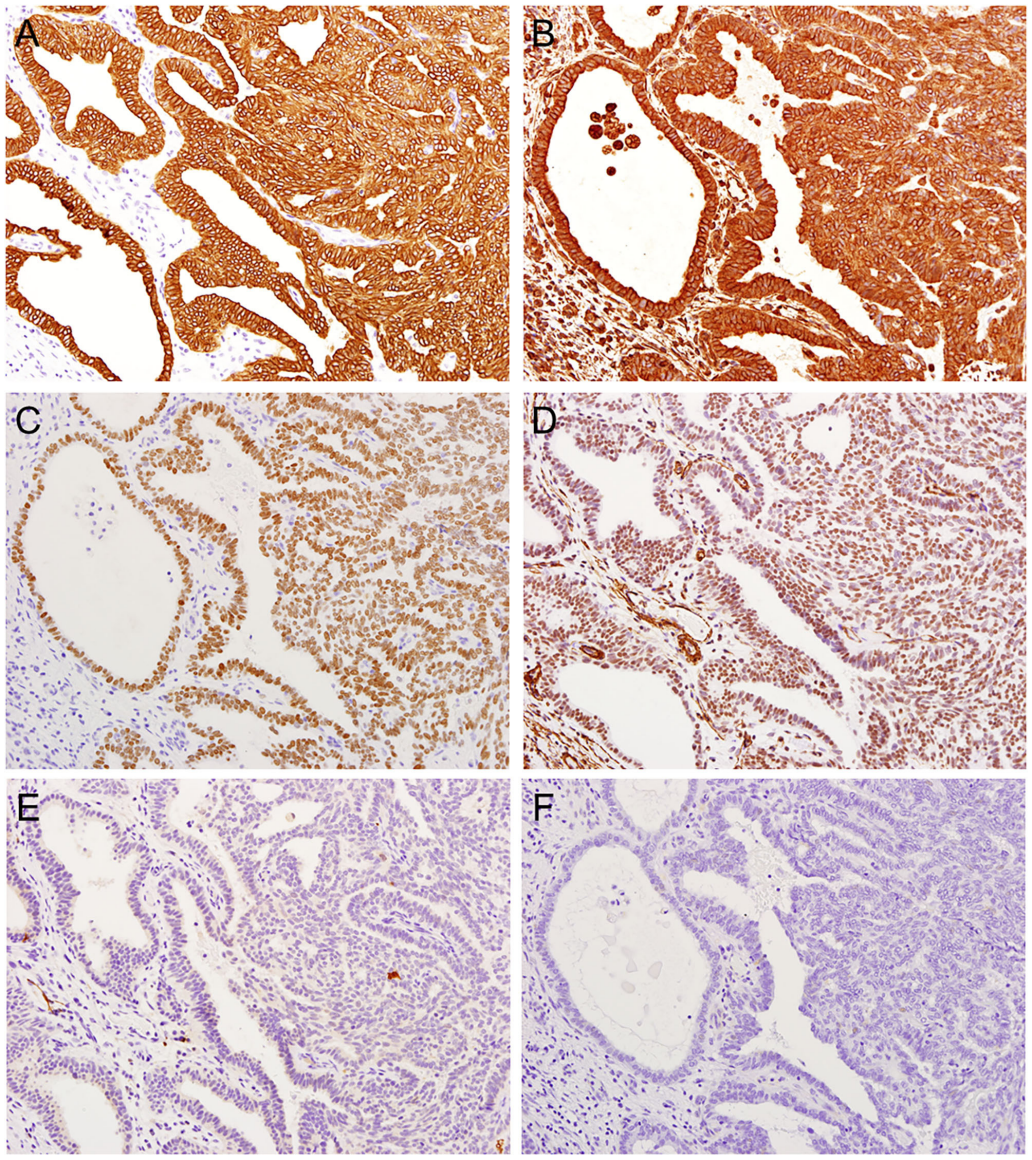
Immunohistochemical features of TL-LGNPPA. **(A)** Positive staining for cytokeratin (CK) AE1/AE3 (× 20). **(B)** Positive staining for vimentin (× 20). **(C)** Positive nuclear staining for thyroid tissue factor-1 (TTF-1) (× 20). **(D)** Negative staining for smooth muscle actin (SMA) (× 20). **(E)** Negative staining for S100 (× 20). **(F)** Negative staining for thyroglobulin (× 20).

Immunohistochemistry revealed that the tumor cells were positive for cytokeratin (CK) AE1/AE3 (Figure 3A), CK7, CK19, epithelial membrane antigen (EMA), vimentin (Figure 3B), and thyroid tissue factor-1 (TTF-1) (Figure 3C) but negative for CK5/6, CK20, smooth muscle actin (SMA) (Figure 3D), calponin, p63, glial fibrillary acidic protein (GFAP), S100 (Figure 3E), CDX2, CEA, PAX8, CD10, DOG1, GATA3, SOX10, GCDFP-15, and thyroglobulin (Figure 3F). A pathological diagnosis of LGNPPA with TTF-1 (i.e., TL-LGNPPA) was finally made. The surgical margin was negative. Adjuvant therapy was not performed because of the free histopathological margin and information about the clinical characteristics of TL-LGNPPA reported in the relevant literature. There was no evidence of recurrence or distant metastasis at 5 years after surgery. The patient is currently being followed up and is satisfied with the good clinical course and lack of post-treatment symptoms.

## Review of the Relevant Literature on TL-LGNPPA

We identified only 27 case reports of TL-LGNPPAs or LGNPPAs with the expression of TTF-1 that included detailed patient information in 23 English-language articles published up to 2019 (5–26). We reviewed the relevant literature on TL-LGNPPAs or LGNPPAs with the expression of TTF-1 in terms of the clinicopathological and immunohistochemical characteristics, including our case (Tables 1, 2).

**Table 1:** Summary of clinical characteristics of previously reported thyroid-like low-grade nasopharyngeal papillary adenocarcinoma.

	**Age**	**Sex**	**Symptoms**	**Duration of symptoms**	**Site**	**Size**	**Nature of tumor**	**Preoperative biopsy and pathological diagnosis**	**Treatment**	**Follow up**	**Adjuvant therapy**	**Metastasis or Relapse**
Carrizo and Luna (5)	9	M	Right nasal fullness, blood in his saliva	3 m	The right nasopharyngeal wall	2.0 cm	Submucosal mass focal erosion	+; LGNPPA	A transpalatal resection	2 y	No	No
Carrizo and Luna (5)	13	M	Unilateral nasal obstruction	2 m	The roof of the nasopharynx at the junction of the nasal septum and the vault	1.5 cm	Mass	+; LGNPPA	A transpalatal resection	15 y	No	No
Wu et al. (8)	36	F	No symptom (discovered incidentially)	0 d	The left roof of the nasopharynx	1 cm	A yellowish polypoid mass	No	Complete endoscopic excision	3 y	No	No
Fu et al. (9)	68	M	A globus sensation in his throat	2 w	The roof of the nasopharynx	ND	Pedunculated tumor with its stalk	+; papillary adenocarcinoma in the nasopharynx	Completely resection by facial translocation approach	1 y	No	No
Ohe et al. (10)	25	M	Bloody sputum	3 m	The roof of the pharynx	0.8 cm	Pedunculated polypoid mass	No	Endoscopic excision	13 m	No	No
Ohe et al. (10)	41	M	No symptom (discovered incidentially)	0 d	The posterior roof of the nasopharynx	0.5 cm	Polypoid mass	+; TL-LGNPPA	Complete endoscopic excision	9 m	No	No
Sillings et al. (11)	19	M	Intermittent bilateral epistaxis, nasal congestion	Several months	The posterior superior free edge of the nasal septum	~1.5 cm	Soft tissue mass that was pink, freely mobile, and pedunculated	No	Endoscopic excision	ND	No	No
Petersson et al. (12)	39	F	Right-sided epistaxis, frequent blocked nose, rhinorrhea	4 m	The posterior edge of the bony septum	1 cm	A polypoidal mass	+; Low-grade NPAC	Endoscopic resection	ND	No	No
Chu et al. (13)	50	M	Blood-tinged rhinorrhea, morning headache	10 d	Left nasopharynx	7 mm	The exophytic nasopharyngeal mass	+; a low-grade NPAC	A nasopharyngectomy using a diode laser	3 m	No	No
Ozer et al. (14)	17	F	Nasal obstruction	6 m	The posterior nasopharyngeal wall	27 × 22 mm	Large, bilobulated nasopharyngeal mass	+; low grade NPAC	Endoscopic excision	1 y	No	No
Ryu et al. (15)	31	F	Nasal obstruction, mild postnasal drip	Several weeks	The narrow area around the cranial end of the nasal septum and the nasopharyngeal vault	Completely occluding nasopharynx	Exophytic, irregularly-surfaced, and very fragile tumor	+; LGNPPA	Exclusive endoscopic resection	3y	No	No
Oishi et al. (7)	47	F	Nasal obstruction	ND	Posterior edge of the left nasal septum	2.0 cm	A pedunculated, hemorrhagic mass	+; TL-LGNPPA	Resection	19 m	No	No
Huang et al. (16)	36	F	Right epistaxis persistent nasal obstruction	3 m	The roof of the nasopharynx	ND	A pedunculated tumor	No	Complete Endoscopic excision	31 m	No	No
Ozturk et al. (17)	24	F	Nasal congestion	1 y	The posterior septum	~3.0 × 2.5 cm	Mass with a smooth surface	No	Complete endoscopic excision	4 y	No	No
Borsetto et al. (18)	15	F	The sudden onset of posterior nose bleeding	0 d	Bony ridge of the vomer	ND	A pedunculated vegetating nasopharyngeal lesion	+; TL-LGNPPA	A type II nasopharyngeal endoscopic resection	30 m	No	No
Horino et al. (19)	25	F	Fever of unknown origin	2 y	Nasopharyngeal lesion	1.7 × 1.2 cm	The pedunculated mass	No	Complete resection	3 y	No	No
Rajeswari et al. (20)	13	M	Nasal obstruction mild epistaxis	2 m	Nasopharynx	ND	A polypoidal lesion	No	Surgical excision	1 y	No	No
Li et al. (21)	15	F	Rhinorrhoea nasal congestion	1 m	The posterior nasal septum	~2.5 × 2 cm	A pedunculated polypoid mass with smooth surface	+; adenocarcinoma with papillary structure	Endoscopic complete excision	2 y	No	No
Oide et al. (22)	68	M	Sore throat hemosputum	2 w	On the roof of the nasopharynx	8 × 4 mm	A dark red polypoid mass	No	Endoscopic resection	ND	No	No
Yang et al. (23)	27	F	Frequent blocked nose rhinorrhea, mild headache	2 y	The posterior edge of the nasal septum	1.8 cm	Mass	+; TL-LGNPPA	The removal of the whole mass	3 y	No	No
Yang et al. (23)	34	F	Tinnitus and loss of hearing	4 d	Top of nasopharynx	0.5 cm	A nodular mass	No	The removal of the whole mass	1 y	No	No
Yang et al. (23)	23	M	Nasal discomfort	3 d	The back end of nasopharyngeal roof	0.5 cm	A polypoidal tumor	No	The removal of the whole mass	1 y	No	No
Zhang et al. (24)	64	M	Nasal bleeding, a foreign body sensation within the nasopharynx	ND	The posterior wall of the nasopharynx	2.0 cm	Broad-based mass with a smooth surface	+; TL-LGNPPA	Complete surgical resection	12 m	No	No
Baumann and Betz (27)	26	M	Nasal congestion, epistaxis.	ND	The junction of the posterior nasal cavity and nasopharynx	ND	Polypoid mass with a distinctly papillary proliferation	No	Resection	ND	No	No
Ünsaler et al. (25)	9	M	Nasal obstruction, sleep apnea, nasal bleeding	6 m	The roof to the posterior nasopharyngeal wall	ND	Polypoid residual tissue	+ (adenoidectomy); papillary adenocarcinoma	An endoscopic posterior septectomy, the additional entire resection using fibered thulium-YAG laser	5y	No	No
Yokoi et al. (26)	58	M	No symptom (discovered incidentially)	0 d	The posterior end of the nasal septal mucosa at the midline of the epipharynx	A diameter of approximately 10 mm	Round tumor	+; an inverted ductal papilloma of the salivary glands	Endonasal endoscopic excisation of tumor	34 m	No	No
Li et al. (6)	35	F	Dyspnea after activities, pharyngeal foreign body sensation of unkown cause dry throat	3 d	The posterior edge of the nasal septum	1.5 × 1.0 × 0.8 cm	Smooth neoplasm with pedicel, smooth soft gray polypoid tumor	No	Complete removal of tumor under the nasopharyngoscope	16 m	No	No
Present case	38	F	Nasal obstruction	1 m	The posterior edge of nasal septum	20 mm	Pedunculated polypoid tumor	+; benign salivary gland type tumor	Endonasal endoscopic excisation of tumor	5 y	No	No

*M, male; F, female; ND, not described; d, days; m, months; y, years; cm, centimeter; mm, millimeter; TL-LGNPPA, thyroid-like low-grade nasopharyngeal papillary adenocarcinoma; LGNPPA, low-grade nasopharyngeal papillary adenocarcinoma; NPAC, nasopharyngeal adenocarcinoma*.

**Table 2:** Summary of characteristics of immunohistochemical and *in situ* hybridization investigations of previously reported thyroid-like low-grade nasopharyngeal papillary adenocarcinoma.

**Report**	**TTF-1**	**CK7**	**CK19**	**vimentin**	**CKpan**	**EMA**	**TG**	**CK20**	**S100**	**CK5/6**	**p63**	**SMA**	**CDX2**	**GFAP**	**CEA**	**PAX8**	**CD10**	**DOG1**	**GATA3**	**SOX10**	**GCDFP-15**	**EBV**	**HPV**
Carrizo and Luna (5)	+	+	+	ND	ND	ND	–	–	ND	–	ND	ND	ND	ND	ND	ND	ND	ND	ND	ND	ND	ND	ND
Carrizo and Luna (5)	+	+	+	ND	ND	ND	–	–	ND	–	ND	ND	ND	ND	ND	ND	ND	ND	ND	ND	ND	ND	ND
Wu et al. (8)	+	+	ND	ND	ND	ND	–	–	ND	ND	ND	ND	ND	ND	ND	ND	ND	ND	ND	ND	ND	–	–
Fu et al. (9)	+	+	ND	ND	ND	ND	ND	–	ND	ND	ND	ND	ND	ND	ND	ND	ND	ND	ND	ND	ND	–	ND
Ohe et al. (10)	+	+	+	+	ND	ND	–	–	–	–	ND	ND	ND	ND	ND	ND	ND	ND	ND	ND	ND	–	ND
Ohe et al. (10)	+	+	+	+	ND	ND	–	–	–	–	ND	ND	ND	ND	ND	ND	ND	ND	ND	ND	ND	–	ND
Sillings et al. (11)	+	ND	ND	ND	+	ND	–	ND	–	ND	ND	ND	ND	ND	ND	ND	ND	ND	ND	ND	ND	ND	ND
Petersson et al. (12)	+	ND	+	+	+	+	–	ND	–	ND	–	ND	–	ND	ND	ND	ND	ND	ND	ND	ND	ND	ND
Chu et al. (13)	+	ND	ND	ND	+	ND	–	ND	–	ND	ND	ND	ND	ND	ND	ND	ND	ND	ND	ND	ND	ND	ND
Ozer et al. (14)	+	+	+	ND	ND	ND	+ (focal)	ND	ND	ND	ND	ND	ND	ND	ND	ND	ND	ND	ND	ND	ND	ND	ND
Ryu et al. (15)	+	+	ND	ND	ND	ND	–	–	ND	ND	ND	ND	ND	ND	ND	ND	ND	ND	ND	ND	ND	ND	ND
Oishi et al. (7)	+	+	+	+	+	ND	–	–	–	–	–	–	ND	ND	ND	–	ND	ND	ND	ND	ND	ND	ND
Huang et al. (16)	+	ND	ND	ND	ND	ND	–	ND	ND	ND	ND	ND	ND	ND	ND	ND	ND	ND	ND	ND	ND	ND	ND
Ozturk et al. (17)	+	ND	ND	ND	ND	ND	–	ND	ND	ND	ND	ND	ND	ND	ND	ND	ND	ND	ND	ND	ND	ND	ND
Borsetto et al. (18)	+	+	ND	ND	+	ND	ND	ND	ND	ND	ND	ND	ND	ND	ND	ND	ND	ND	ND	ND	ND	–	–
Horino et al. (19)	+	+	ND	+	ND	ND	–	–	–	–	–	–	ND	ND	ND	ND	ND	ND	ND	ND	ND	ND	ND
Rajeswari et al. (20)	+	+	ND	ND	ND	ND	–	–	ND	ND	ND	ND	ND	ND	ND	ND	ND	ND	ND	ND	ND	ND	ND
Li et al. (21)	+	ND	ND	+	+	+	–	–	–	ND	ND	ND	–	–	ND	ND	ND	ND	ND	ND	ND	–	ND
Oide et al. (22)	+	+	+	+	+	+	–	–	–	+	+	–	ND	–	ND	–	ND	ND	ND	ND	ND	–	ND
Yang et al. (23)	+	+	+	+	+	+	–	ND	–	ND	ND	ND	ND	ND	–	ND	ND	ND	ND	ND	ND	–	ND
Yang et al. (23)	+	+	+	+	+	+	–	ND	–	ND	ND	ND	ND	ND	–	ND	ND	ND	ND	ND	ND	–	ND
Yang et al. (23)	+	+	+	+	+	+	–	ND	–	ND	ND	ND	ND	ND	–	ND	ND	ND	ND	ND	ND	–	ND
Zhang et al. (24)	+	+	+	ND	ND	ND	–	–	–	–	–	ND	–	ND	ND	–	ND	ND	ND	ND	ND	–	–
Baumann and Betz (27)	+	+	ND	ND	ND	+	–	ND	ND	–	ND	ND	ND	ND	ND	ND	ND	ND	ND	ND	ND	ND	ND
Ünsaler et al. (25)	+	+	ND	ND	ND	ND	–	–	–	ND	–	ND	–	ND	ND	ND	ND	ND	ND	ND	ND	ND	ND
Yokoi et al. (26)	+	+	+	+	ND	ND	–	–	–	ND	–	–	ND	ND	ND	ND	ND	ND	ND	ND	ND	ND	ND
Li et al. (6)	+	+	+	+	+	ND	–	–	–	ND	–	–	–	–	ND	ND	ND	ND	ND	ND	ND	ND	ND
Present case	+	+	+	+	+	+	–	–	–	–	–	–	–	–	–	–	–	–	–	–	–	–	ND
Number of cases performed exam	28	22	15	13	12	8	26	17	17	10	9	6	6	4	4	4	1	1	1	1	1	12	3
posive or negative ratio	+; 100%	+; 100%	+; 100%	+; 100%	+; 100%	+; 100%	–; 96,2%	–; 100%	–; 100%	–; 90.0%	–; 88.8%	–; 100%	–; 100%	–; 100%	–; 100%	–; 100%	–; 100%	–; 100%	–; 100%	–; 100%	–; 100%	–; 100%	–; 100%

*CK, cytokerarin; EMA, Epithelial Membrane Antigen; EBV, Epstein-Barr virus; GFAP, glial fibrillary acidic protein; HPV, human papilloma virus; CEA, carcinoembryonic antigen; SMA, smooth muscle actin; TG, thyroglobulin; TTF-1, thyroid transcription factor-1; +, positive, –, negative; ND, not described*.

### Clinicopathological Characteristics of TL-LGNPPA

In the 28 case reports included in our literature review (Table 1), the mean age of patients with TL-LGNPPA was 32.3 years (range: 9–68 years). Fourteen of the patients were men and 14 were women; thus, the incidence of TL-LGNPPA did not differ according to sex. The clinical symptoms of the patients included nasal obstruction or nasal congestion (*n* = 14, 50.0%), bleeding (including epistaxis and bloody sputum; *n* = 12, 42.9%), nasal or pharyngeal discomfort (including foreign body sensation; *n* = 5, 17.9%), rhinorrhea (*n* = 3, 10.7%), and headache (*n* = 2, 7.1%). Three patients had no clinical symptoms (*n* = 3, 10.7%). Sleep apnea, dry throat, postnasal drip, dyspnea, sore throat, prolonged fever for 2 years, and loss of hearing were also found in one case each (3.6%). Regarding the duration of symptoms, 1 year was defined as 12 months and 1 month was defined as 30 days when the duration was indicated in years and months, respectively. Several weeks and several months were defined as 3 weeks and 3 months, respectively. The mean and median duration of symptoms was 116.6 and 30 days (range, 0–730 days), respectively. The mean maximum tumor diameter was 14.8 mm (range, 5–30 mm).

Regarding appearance of the tumor, the TL-LGNPPAs tended to be pedunculated (*n* = 10, 35.7%) and polypoid (*n* = 12, 42.9%) tumors. The tumor locations included the posterior edge of the nasal septum (*n* = 10, 35.7%), the roof or vault of the nasopharynx (*n* = 9, 32.1%), the narrow area around the cranial end of the nasal septum and the nasopharyngeal vault (*n* = 3, 10.7%), and the posterior wall of the nasopharynx (*n* = 2, 7.1%). The tumor was located in the right or left wall of the nasopharynx in one case each (3.6%). Incisional biopsy was performed before treatment in 16 cases. TI-LGNPPA or LGNPPA was histopathologically diagnosed in 14 patients (87.5%); however, 2 patients including our case were diagnosed as having benign tumors.

All cases were treated with complete resection and without adjuvant treatment. Endoscopic surgery was performed in 16 cases (57.1%). Transpalatal and facial translocation approaches were used in 2 and 1 case, respectively. No detailed information about the surgical approach in the remaining 9 cases was found in the literature. In four cases, secondary resection was performed due to an insufficient surgical margin after the first operation. No patients received adjuvant therapy after complete resection. The mean follow-up period was 31.0 months [range, 3–148 months (15 years)]. No distant metastasis or recurrence was found in any of the cases.

### Characteristics of Immunohistochemical and *in situ* Hybridization Investigations of TL-LGNPPA

We reviewed the results of the immunohistochemical examinations in the 28 cases of TL-LGNPPA, including our case (Table 2). These examinations indicated the positive expression of CK7 (100%, 22/22 cases), CK19 (100%, 15/15 cases), vimentin (100%, 13/13 cases), CKpan (100%, 12/12 cases), and EMA (100%, 8/8 cases) and the negative expression of thyroglobulin (96.2%, 25/26 cases), CK20 (100%, 17/17 cases), S100 (100%, 17/17 cases), CK5/6 (90.0%, 9/10 cases), p63 (88.8%, 8/9 cases), SMA (100%, 6/6 cases), CDX2 (100%, 6/6 cases), GFAP (100%, 4/4 cases), PAX8 (100%, 4/4 cases), and CEA (100%, 4/4 cases). The expression of CD10, DOG1, GATA3, SOX10, and GCDFP-15 was examined only in our case, and all were negative.

*In situ* hybridization to detect Epstein-Barr Virus (EBV) and human papilloma virus (HPV) was negative in all cases (12/12 cases [100%] and 3/3 cases [100%], respectively).

## Discussion

Primary nasopharyngeal adenocarcinomas (NPACs) can be classified by their morphological features and clinical behavior into 2 main categories: the conventional or mucosal surface origin type and the salivary gland type (3). The former is usually a low-grade tumor with papillary configuration and likely originates from the nasopharyngeal surface mucosa (i.e., LGNPPA), whereas the latter consists of tumors such as mucoepidermoid adenocarcinoma, adenoid cystic carcinoma, and polymorphous low-grade adenocarcinoma (3). TL-LGNPPA is an extremely rare neoplasm that exhibits morphologic features that are analogous to papillary thyroid carcinoma and the abnormal expression of TTF-1.

From our review of the 28 cases reported in the literature (including the present case), most TL-LGNPPAs had a pedunculated and polypoid shape and were located at the vault of epipharynx and the posterior edge of the nasal septum. Patients with TL-LGNPPA were most commonly in their thirties, and there was no sex difference in its incidence. The main symptoms of TL-LGNPPA were nasal obstruction or nasal congestion, bleeding, and nasal or pharyngeal discomfort. In our case, the main symptom was nasal obstruction, which persisted for 1 month after the onset of symptoms of a common cold. Horino et al. (19) reported the case of a 25-year-old woman who had long-lasting fever of unknown origin. They hypothesized that the overexpression of TTF-1 in the tumor might have induced the local expression of interleukin-6, resulting in the fever. Furthermore, it was suggested that TL-LGNPPA has an excellent prognosis and that local invasion or distant metastasis of TL-LGNPPA was extremely rare. Some studies have reported the local extension of LGNPPA into the sphenoid sinus (28) or parapharyngeal space (29); however, an immunohistochemical study was negative for TTF-1 in these two cases of LGNPPA. TL-LGNPPA might tend to grow more slowly than LGNPPA without the expression of TTF-1, and the frequency of local invasion might differ from that in LGNPPA without the expression of TTF-1.

Endoscopic complete resection would be recommended as the first choice of treatment for TL-LGNPPA. In 4 of 28 cases of TL-LGNPPA, additional resection of the tumor was performed after insufficient primary surgery; however, none of these patients developed local recurrence or distant metastasis. In our case, endoscopic resection with a 5-mm safety margin was performed, and 5-year disease-free survival was achieved. Some studies reported cases of LGNPPA treated with adjuvant therapy after complete resection. Wang et al. (28) used photodynamic therapy as a postoperative adjuvant therapy for an incompletely resected primary nasopharyngeal papillary adenocarcinoma and achieved 5-year disease-free survival. In other reports, adjuvant radiotherapy was performed, and no patients developed recurrent disease or distant metastasis (4, 30). These LGNPPAs were negative for TTF-1, or TTF-1 expression was not investigated. In TL-LGNPPA or LGNPPA with TTF-1, serial adjuvant therapy might be excessive after complete resection. We cannot draw any complete conclusions regarding the need for adjuvant therapy due to the small number of case reports of TL-LGNPPA. Further accumulation of cases and long-term follow-up will be necessary.

Histopathologically, TL-LGNPPAs are composed of a complex, arborizing papillary configuration with hyalinized fibrovascular cores and glands lined by cuboidal to columnar cells with a moderate amount of eosinophilic cytoplasm and round to oval nuclei with vesicular to clear chromatin (31). Psammomatoid calcifications are seen in approximately one-third of cases. With the exception of psammoma bodies, most findings were present in our case. The spindle cell component was found in our case. In the previous literature up to 2019, only five cases of TL-LGNPPA with a prominent spindle cell component, named “biphasic low-grade nasopharyngeal papillary adenocarcinoma,” had been reported (7, 10, 12, 26). Whether the etiology of biphasic LGNPPA differs from TL-LGNPPA without the spindle cell component and the reason why it develops in TL-LGNPPA remain unknown. Petersson et al. discussed tumors with a biphasic appearance, such as a biphasic synovial sarcoma, a medullary thyroid carcinoma, or a group of enigmatic neoplasms that show “thymic-like or brachial pouch differentiation” as differential diagnoses of biphasic TL-LGNPPA (12). In their report, biphasic LGNPPA was differentiated from these tumors based on diffuse positivity of CK and no evidence of gene alternations such as SYT-SSX1/2 fusion transcripts t(X; 18) (p11.2; q11.2) against a biphasic synovial sarcoma, the absence of immunopositivity for CEA, calcitonin, and chromogranin A against medullary thyroid carcinoma, and the difference in originating regions or the expression of TTF-1 against a group of enigmatic neoplasms, respectively (12). Only one report mentioned TL-LGNPPA with squamous differentiation (22); however, Yokoi et al. posited that the development of spindle cells occurred independently of squamous differentiation as the tumors were negative for CK5/6 and p40 (26). Further research and accumulation of cases are needed to elucidate the pathogenesis of TL-LGNPPA with the spindle cell component.

Immunohistochemically, the most characteristic finding of TL-LGNPPA is TTF-1 positivity (5). TTF-1 is a member of the homeodomain transcription factor family that regulates genes expressed within the thyroid, lung, and brain, including thyroglobulin, thyroid peroxidase, Clara cell secretory protein, and surfactant proteins (32). TTF-1 is not only found in TL-LGNPPA but also in primary thyroid papillary adenocarcinoma or lung cancer, suggesting that metastatic thyroid papillary adenocarcinoma or lung cancer in the nasopharynx is important as a differential diagnosis of TL-LGNPPA. Immunostaining of thyroglobulin is highly recommended to differentiate TL-LGNPPA from metastatic thyroid papillary adenocarcinoma (6). Thyroglobulin is usually detected in metastatic thyroid papillary adenocarcinoma, whereas TL-LGNPPA is negative for thyroglobulin. These findings were also observed in our case.

As an additional differential diagnosis of TL-LGNPPA, clinicians should also consider low-grade sinonasal adenocarcinoma, which is divided into salivary type, intestinal type, and non-intestinal type adenocarcinoma (33).

The papillary variant of PLGA is one of the most important differential diagnoses of TL-LGNPPA. PLGA is a minor salivary gland neoplasm that is characterized by morphologic variability, cytologic uniformity, and an infiltrating growth pattern (34). In the WHO classification of salivary gland tumors that was updated in 2017, PLGA was renamed and shortened to polymorphous adenocarcinoma (PAC) (35, 36). PLGA may also have a papillary architecture with an invasive growth pattern and slight nuclear atypia; as a result, it is difficult to distinguish PLGA from TL-LGNPPA based on the morphology alone (3, 24). Immunohistochemical examinations are important to distinguish PLGA from TL-LGNPPA. PLGA (PAC, classical variant) is positive for CK7, CK8, CK18, vimentin, p63 (a myoepithelial marker), and S100 and negative for CK19, p40, and TTF-1 (3, 36). In our case, PLGA and LGNPPA were suggested as differential diagnoses based on the morphology; however, immunohistochemistry was negative for p63 and S100 and positive for TTF-1; thus, the final diagnosis was TL-LGNPPA.

The latest WHO classification of salivary gland tumors includes under the PAC heading not only classical PLGA but also the so-called “cribriform adenocarcinoma of minor salivary glands” (CAMSG) (35, 36). Clinically, CASMG often shows more aggressive regional and distant spread compared to PLGA (PAC, classical variant) (35, 36). Microscopically, the tumor cells of CAMSG show pale and vesicular nuclei with a ground-glass appearance that resembles the Orphan Annie Eye-nuclei of papillary thyroid carcinoma (PTC) (36). CAMSG shows architectural uniformity with a predominant cribriform and solid growth pattern, sometimes with peripheral palisading, peripheral clefting, and glomeruloid appearance (37). Immunohistochemically, CAMSG is strongly positive for CK7, CK8, CK18, S100, and vimentin. Furthermore, AE1/AE3 and SOX10 are strongly expressed in CAMSG (36). Basal and myoepithelial markers such as p63, SMA, and CK5/6 are variably positive in all CASMGs. As with PAC, CAMSG is also consistently negative for p40. CK19 stains in most CAMSG although in a mild-to-moderate manner (36). Most importantly, CAMSG is consistently negative for both thyroglobulin and TTF-1 (38). Despite the histologic and immunophenotypic similarity between PLGA and CAMSG, partially different genetic alterations in PRKD genes were found in PLGA and CAMSG (36). More than 70% of PACs exhibit activating mutations in the PRKD1 gene, which is a single-nucleotide variant (E710D) that affects a highly conserved amino acid in the catalytic loop of the kinase domain (36). In contrast to PAC, 80% of CAMSGs show rearrangements rather than mutations in PRKD genes (PRKD1-3) (36). From our review of the literature and our case, we could not find any results of the examination of PRKD gene alteration including in our case. However, we could also differentiate CAMSG in the final diagnosis because of the difference in immunohistochemical findings in our case (i.e., positive for TTF-1 and negative for S100, CK5/6, p63, SMA, and SOX10) despite that lack of PKRD studies.

Papillary variants of intestinal-type adenocarcinoma (ITAC) should also be excluded in the diagnosis of TL-LGNPPA (6). ITACs comprise a significant proportion of primary adenocarcinomas of the sinonasal tract, which show histological features that are reminiscent of colonic adenoma and adenocarcinoma (39, 40). ITACs are characteristically associated with occupational exposure to wood and leather dust, and have more aggressive behavior, characterized by repeated local recurrence and an ominous outcome in comparison to low-grade papillary adenocarcinoma (39, 40). Immunohistochemically, ITACs are positive for the expression of CK7, CDX-2, villin, MUC2, SATB-2, and CK20, whereas TL-LGNPPAs are negative for CDX-2 and CK20 (39).

Sinonasal renal cell-like adenocarcinoma (SNRLCA) is a variant of low-grade, non-intestinal sinonasal adenocarcinoma that mimics renal cell carcinoma (41). This entity histologically resembles clear cell renal cell carcinoma, with nests and follicles of polyhedral cells with abundant optically clear cytoplasm (41). Nuclear pleomorphism and mitotic activity are minimal (41). Immunochemically, it has been reported to be positive for CK7 and carbonic anhydrase IX (CAIX) and negative for PAX8, RCC, and vimentin (41, 42). The expression of S100 is variable and inconsistent in SNRLCA (42). Some case reports of SNRCLA indicated positive expression for DOG1 and SOX 10 (43, 44). Positivity for S100, DOG1, and SOX10, which are markers of seromucinous differentiation, might depend on the degree of seromucinous differentiation of SNRLCA (33, 44). Other reports indicated that TTF-1 was completely negative in SNRCLA (43–46). We could not find any examinations of CAIX from our review of the literature including our case, but SNRCLA could be differentiated in the final diagnosis because our case was positive for TTF-1 and vimentin.

ETV6 rearranged sinonasal low-grade non-intestinal type adenocarcinoma is one of the differential diagnoses of TL-LGNPPA. Three cases of this novel entity restricted to the sinonasal tract were first reported by Andreasen et al. in 2017 (47). Morphologically, this tumor is tubular, composed of cuboidal to cylindrical tumor cells with bright eosinophilic cytoplasm, which is positive for periodic acid-Schiff with diastase in the atypical portion. Nuclei are basally located, compact, or vesicular with granular chromatin and a single centrally located nucleoli. Tumor stroma is sparse, but fine fibrovascular strands are seen between tubular formations. Mitoses are rare, and no vascular or perineural growth is observed. Immunohistochemically, the tumor cells are positive for CK7, SOX10, DOG1, vimentin, S100, and GCDFP-15 and negative for CK20, GATA3, and mammaglobin (47, 48). This entity is characterized by the gene rearrangement of ETC variant 6 (ETV6), which is a fusion between ETV6 and the Neurotrophic receptor tyrosine kinase type 3 (NTRK3) or RET gene identified with fluorescence *in situ* hybridization (FISH) (47, 48). FISH for ETV6 rearrangement in previous reports was not performed in our case or in the previous literature for TL-LGNPPA. However, we could differentiate TL-LGNPPA from ETV6 rearranged sinonasal low-grade non-intestinal type adenocarcinoma on the basis of the immunohistochemical findings in our case (i.e., positive for TTF-1 and negative for S100, CK5/6, DOG1, SOX10, and GCDFP-15).

Nasopharyngeal carcinoma has been shown to be strongly associated with Epstein-Barr virus (EBV) (49). *In situ* hybridization reveals that EBV-encoded RNA (EBER) is strongly expressed by the tumor cells in these cases, indicating the presence of EBV RNA (50). From our review of the literature, all 12 cases of TL-LGNPPA, including our case and the cases reported in 8 studies in the literature, were completely negative for EBER (8, 9, 11, 18, 21–24). Furthermore, 3 cases reported in 3 studies in the literature were negative for human papilloma virus (HPV) (5, 18, 24). These findings suggest that the origin of TL-LGNPPA might not be associated with EBV or HPV infection; however, further investigations are needed due to the relatively small number of cases.

Gene alterations of TL-LGNPPA were only analyzed in 3 cases in 3 studies (7, 12, 22). BRAF V600E mutations, the most common gene alteration in PTC, were not detected in any of the 3 reported cases of TL-LGNPPA in which genetic analyses were performed. Oide et al. also performed sequencing for mutations in *N-RAS* (codon 61) genes, which are known to be mutated in PTC, in a case of NPAC (22). However, the results were negative, and they concluded that although morphologically similar, NPAC and PTC do not share the same molecular pathogenesis (22). Furthermore, they analyzed the EGFR and ALK genes, which are involved in some populations of TTF-1-positive lung adenocarcinoma, and found no mutations (22). Petersson et al. investigated BRAF and KIT mutations and SYT-SSX1/2 rearrangement, but they did not identify any particular genetic alterations (12). As shown in these reports, no specific gene alterations have been found in TL-LGNPPA, and the tumorigenesis of TL-LGNPPA remains unclear. We summarize the differentiation of immunohistochemical findings and gene alternations between TL-LGNPPA and other sinonasal adenocarcinomas in Table 3 (33–48, 51–56). The accumulation of more cases and further investigations will be needed to elucidate the pathogenesis of this rare neoplasm.

**Table 3:** Differentiation between TL-LGNPPA and other sinonasal adenocarcinomas [Refer to (33–48, 51–56)].

	**Immunohistochemical findings**																	**Gene profile**
	**TTF-1**	**TG**	**CK7**	**CK19**	**CK20**	**Vimentin**	**EMA**	**S100**	**CK5/6**	**p63**	**SMA**	**PAX8**	**DOG1**	**GATA3**	**SOX10**	**GCDFP-15**	**Other specific markers**	
TL-LGNPPA	+	–	+	+	–	+	+	–	–	–	–	–	–	–	–	–	ND	Negative for alterations of BRAF V600E, N-RAS, EGFR, ALK, and SYT-SSX1/2 genes
Metastatic papillary thyroid carcinoma	+	+	+	+	+/–	ND	ND	ND	ND	ND	ND	+	ND	ND	ND	ND	Galectin-3, HBME-1	RET oncogene BRAF V600E mutation
Metastatic lung adenocarcinoma	+	–	+	ND	–	+/–	ND	ND	–	+/–	ND	ND	ND	ND	ND	ND	Napsin A	Tyrosine kinase inhibitors, ALK, ROS1, EGFR genes
PLGA (PAC classical variant)	–	–	+	–	ND	+	ND	+	ND	+	+/–	ND	+	ND	ND	ND	ND	Mutation in the PRKD1 gene (single-nucleotide variant (E710D))
CAMSG	–	–	+	+	ND	+	–	+	+/–	+/–	+/–	ND	ND	ND	ND	ND	ND	Rearrangement of PKRD gene (PKRD1-3) including ARIDIA-PRKD1 and DDX3X-PRKD1 gene fusion
ITAC	ND	ND	+	ND	+	ND	ND	ND	ND	ND	ND	ND	ND	ND	ND	ND	CDX2, villin, MUC2, STAB2	TP53 mutation, CDKN2A alteration, variable β-catenin expression
SNRCLA	–	–	+	–	–	–	+	+/–	ND	ND	ND	–	+/–	–	+/–	ND	CAIX	ND
ETV6 rearranged SNLGAC	–	–	+	ND	–	+/–	ND	+	ND	ND	ND	ND	+	+/–	+	+	ND	ETV6-NTRK or ETV6-RET fusion

*TL-LGNPPA, thyroid-like low-grade papillary adenocarcinoma; +/–, variably positive; +, positive; –, negative; ND, not described; PLGA, polymorphous low-grade adenocarcinoma; PAC, polymorphous adenocarcinoma; CAMSG, cribriform adenocarcinoma of minor salivary glands; ITAC, intestinal type adenocarcinoma; SNRCLA, sinonasal renal cell-like adenocarcinoma; SNLGAC, sinonasal low-grade adenocarcinoma*.

## Conclusion

We treated an extremely rare case of TL-LGNPPA originating from the posterior edge of the nasal septum. From our experience and a review of the literature, TL-LGNPPA has a very good prognosis, and endoscopic complete resection would be recommended as the first-line treatment. In addition to the morphological characteristics, immunohistochemistry is important for the diagnosis, including the differential diagnosis, as TL-LGNPPA is immunohistochemically positive for CK7 and TTF-1 and negative for thyroglobulin, CK20, and S100. Clinicians should pay attention to the possibility of this rare entity if they detect a pedunculated or polypoid mass in the nasopharynx.

## Data Availability Statement

The original contributions presented in the study are included in the article/supplementary materials, further inquiries can be directed to the corresponding author/s.

## Ethics Statement

Ethical review and approval was not required for the study on human participants in accordance with the local legislation and institutional requirements. The patients/participants provided their written informed consent to participate in this study.

## Author Contributions

HTak, TH, HTac, AN, HM, MS, and HS: conception and design. HTak, TH, HM, MS, and HS: literature search and obtaining of images. HTac, TH, MS, and HS: writing the article. All authors: critical revision and final approval of the article.

## Conflict of Interest

The authors declare that the research was conducted in the absence of any commercial or financial relationships that could be construed as a potential conflict of interest.

## Abbreviations

CAIX: carbonic anhydrase IX
CAMSG: cribriform adenocarcinoma of minor salivary glands
CK: cytokeratin
CT: computed tomography
EBER: EBV encoded RNA
EBV: Epstein-Barr virus
EMA: epithelial membrane antigen
GFAP: glial fibrillary acidic protein
HPV: human papilloma virus
ITAC: intestinal-type adenocarcinoma
LGNPPA: low-grade nasopharyngeal papillary adenocarcinoma
MRI: magnetic resonance imaging
NPAC: nasopharyngeal adenocarcinoma
PAC: polymorphous adenocarcinoma
PLGA: polymorphous low-grade adenocarcinoma
PTC: papillary thyroid carcinoma
SMA: smooth muscle actin
SNRLCA: sinonasal renal cell-like adenocarcinoma
TL-LGNPPA: thyroid-like low-grade nasopharyngeal papillary adenocarcinoma
TTF-1: thyroid transcription factor-1.
